# Identification and analysis of genomic islands in *Burkholderia cenocepacia* AU 1054 with emphasis on pathogenicity islands

**DOI:** 10.1186/s12866-017-0986-6

**Published:** 2017-03-27

**Authors:** Feng-Biao Guo, Lifeng Xiong, Kai-Yue Zhang, Chuan Dong, Fa-Zhan Zhang, Patrick C.Y. Woo

**Affiliations:** 1 School of Life Science and Technology, University of Electronic Science and Technology of China, Chengdu, 610054 China; 2 Center for Informational Biology, University of Electronic Science and Technology of China, Chengdu, 610054 China; 3 Key Laboratory for Neuro-information of the Ministry of Education, University of Electronic Science and Technology of China, Chengdu, 610054 China; 4 Department of Microbiology, The University of Hong Kong, Hong Kong, Special Administrative Region People’s Republic of China

**Keywords:** Genomic Island, Pathogenicity Island, *B. cenocepacia* AU1054, Virulence factor

## Abstract

**Background:**

Genomic islands (GIs) are genomic regions that reveal evidence of horizontal DNA transfer. They can code for many functions and may augment a bacterium’s adaptation to its host or environment. GIs have been identified in strain J2315 of *Burkholderia cenocepacia*, whereas in strain AU 1054 there has been no published works on such regions according to our text mining and keyword search in Medline.

**Results:**

In this study, we identified 21 GIs in AU 1054 by combining two computational tools. Feature analyses suggested that the predictions are highly reliable and hence illustrated the advantage of joint predictions by two independent methods. Based on putative virulence factors, four GIs were further identified as pathogenicity islands (PAIs). Through experiments of gene deletion mutants in live bacteria, two putative PAIs were confirmed, and the virulence factors involved were identified as *lipA* and *copR*. The importance of the genes *lipA* (from PAI 1) and *copR* (from PAI 2) for bacterial invasion and replication indicates that they are required for the invasive properties of *B. cenocepacia* and may function as virulence determinants for bacterial pathogenesis and host infection.

**Conclusions:**

This approach of *in silico* prediction of GIs and subsequent identification of potential virulence factors in the putative island regions with final validation using wet experiments could be used as an effective strategy to rapidly discover novel virulence factors in other bacterial species and strains.

**Electronic supplementary material:**

The online version of this article (doi:10.1186/s12866-017-0986-6) contains supplementary material, which is available to authorized users.

## Background

*B. cenocepacia* is one of the major pathogens infecting patients with cystic fibrosis (CF) as well as some other chronic respiratory diseases such as bronchiectasis [1–3]. As an opportunistic pathogen, it can cause chronic lung infections in these patients. *B. cenocepacia* belongs to the *Burkholderia cepacia* complex (BCC), which consists of at least 17 genetically distinct but phenotypically similar species [4]. *B. cenocepacia* was initially the species most commonly isolated from patients with CF, although almost all BCC species have now been isolated from CF populations [5, 6]. By using *recA* gene sequence analysis and multilocus sequence typing, *B. cenocepacia* may be subdivided into four phylogenetic clusters, IIIA to IIID [7]. However, almost all clinically relevant isolates belong to the IIIA and IIIB groups [8, 9]. Epidemiological studies showed that strains ET-12 and several other epidemic dominant in Canada and Europe are part of the IIIA subgroup [10]. In comparison, the dominant epidemic clones in the USA belong to subgroup IIIB [11].

With thousands of sequenced bacterial genomes, there are (at the time of writing: December, 2014) seven assembled genomes from the *B. cenocepacia* species (ftp://ftp.ncbi.nlm.nih.gov/genomes/archive/old_refseq/Bacteria/): strains J2315, H111, H2424, MC0-3, AU1054, DDS 22E-1 and DWS 37E-2 [12]. Without exception, all strains possess three chromosomes of unequal sizes. Since sequences of these seven genomes were completely published, they have been extensively used in many comparative genomics and computational genomic studies [13–19]. For example, we reported that the AU 1054 strain has a distinct gene distribution regarding the most important genes, i.e. essential protein-coding genes and tRNA genes [20]. Its third chromosome contains a higher number of these genes than the larger chromosome II [20]. However, this pattern is absent in the other strains and results from segment translocation between chromosomes I and III [20]. Due to the fact that large-scale translocation has been reported in very few bacteria, this work was often listed as one type of example of chromosome translocation in bacterial genomes [21, 22]. In addition, genomic islands (GIs) have been extensively investigated in the strain J2315 [23]. A total of 14 GIs were revealed in this strain and these GIs occupied 9.3% of its 8.06 Mb chromosome. Interestingly, none of them were found as conserved entities in the two available genomes of *B. cenocepacia* IIIB strains, AU1054 and HI2424 [23].

To further understand genome plasticity and reveal potential pathogenicity islands (PAIs), we identified and analyzed GIs in the strain AU 1054. Consequently, 21 GIs were identified through combining multiple methods. These GIs occupied 7.26% of the complete genome. GIs usually exhibit specific characteristics [24, 25]. First, GIs, particularly those recently inserted, tend to have a distinct composition to that of the host genome, and this feature is generally measured by G + C base deviation. Second, transposases and integrases, as mobility genes, may aid host incorporation of the GIs [25, 26] and hence many GIs contain high proportions of mobility genes. Third, tRNA genes, as another type of marker gene [27], often flank GI borders [25, 26]. Fourth, a recent study found that GIs contain higher ratio of hypothetical proteins (predicted proteins with unknown functions) than the rest of the genome [28]. Furthermore, virulence genes more frequently appear in GI regions. Analyses of these features in the 21 putative GIs indicated that they constituted reliable predictions given that each of them was found to contain multiple typical features. Moreover, four GIs were determined as PAIs since they contain putative or recognized virulence factors.

## Methods

### *B. cenocepacia* genomes

Eight strains of *B. cenocepacia* were employed in this work: these are AU 1054, J2315, H2424, HI11, MC0-3, DDS 22E-1, DWS 37E-2 and K56-2. Of these, only the genome of K56-2 has not been sequenced [12]. Sequence data and annotation information of the seven sequenced strains were downloaded from the ftp site of NCBI Refseq in June 2014 [12]. Each genome contains three chromosomes, named I, II and III based on descending order of sequence length. Note that there exist seven other sequenced genomes of this species, but these sequences remain as highly separated fragments and have not been assembled. Therefore, we could not analyze these in a whole chromosome mode as our methods required.

### The cumulative GC profile

The cumulative GC profile method proposed by Zhang and Zhang [29] was used to identify GIs in dozens of prokaryotic genomes [30–33]. Briefly, a chromosomal sequence is projected into a curve called a cumulative GC profile, after the Z transform, linear fitting, and noise filtering [28]. Three basic characteristics of the curve should be indicated: (i) if a region in the curve is almost a straight line, the GC content remains nearly constant within this region. (ii) An elevation (or a decrease) in the profile indicates a reduction (or increase) in GC content. (iii) Any maximum (minimum) point in the curve indicates a turning point, where the GC content undergoes an abrupt change from a relatively GC-poor (GC-rich) region to a relatively GC-rich (GC-poor) region [28].

GIs are typically relatively homogeneous in terms of GC variation, and this fact implies that its curve appears as a straight line [28–33]. According to this characteristic, we could roughly identify candidate GIs by inspection using human eye. To ensure accurate results, an additional index named ‘h’ is employed [28], which describes the homogeneity of GC content of GIs more accurately. If h is significantly less than 1, the variations of GC content of GIs may be considered to be small [33]. In this work, h = 0.1 is taken as the threshold for deciding a potential GI. For more details of the systematic method, please refer to Zhang and Zhang [29].

### The IslandViewer web tool

‘IslandViewer’ is a freely available web tool for predicting GIs [34, 35] and has been widely utilized in identification and characterization of bacterial GIs [36]. For thousands of sequenced bacteria, the web site offers detailed information about their GIs. For an anonymous genome, the tool can perform an automatic search for GIs through composition bias-based methods or comparative genomic approaches. In addition to predictions from cumulative GC profiles, we also downloaded GI information for the AU 1054 strain from IslandViewer.

### Combining the cumulative GC profile and the IslandViewer web tool to obtain reliable predictions

To minimize false-positive predictions, we only retained those GIs predicted by both the cumulative GC profiles and IslandViewer. Such results would have fewer false-positive predictions, although a few actual GIs may be missed by this combinatorial strategy. That is to say, GIs identified by the convergence of the two methods would be more likely to be authentic GIs than those obtained using the individual methods.

### Bacterial strains and growth conditions

The bacterial strains and plasmids used in this study for wet experiments are listed in Table 1. *B. cenocepacia* clinical isolate AU1054 was bought from BCCM-LMBP. Bacteria were routinely cultured in Luria-Bertani (LB) broth with shaking or LB agar plates (LBA) at 37 °C. Unless indicated otherwise, bacterial cultures were supplemented with the following antibiotics (Sigma-Aldrich): ampicillin (Amp, 100 μg/ml), kanamycin (Km, 50 μg/ml), tetracycline (Tet, 30 μg/ml), gentamicin (Gm) (50 μg/ml), trimethoprim (Tp, 50 μg/ml), ceftazidime (Cef, 2 mg/ml) and amikacin (Ami, 2 mg/ml). *B. cenocepacia* cultures were supplemented with 600 μg/ml of Tp and 300 μg/ml of Tet when needed.

**Table 1:** Bacterial strains and plasmids used in gene deletion experiments

Strains or plasmids	Relative characteristics	Source or reference
Strains		
*E. coli* DH5α	F^−^, Ф80d *lacZ*∆M15, ∆(*lacZYA*-*argF*)U169, *endA*1, *recA*1, *hsdR*17(rk^−^, mk^+^) *deoR*, *thi*-1, *supE*44, λ^−^, *gyrA*96(Nal^r^), *relA*1	Invitrogen
*E. coli* SM10(λ pir)	*thi thr leu tonA lacY supE recA*::RP4-2-TC::Mu Km ^r^ λpir	[53]
*B. cenocepacia* AU1054	Clinical isolate	BCCM-LMBP
AU1054*∆copR*	AU1054 derivative with *copR* deletion	This study
AU1054*∆lipA*	AU1054 derivative with *lipA* deletion	This study
Plasmids		
PCRII-TOPO	Cloning vector; *ori lacZ* Km^+^	Invitrogen
pRK2013	*ori*_colE1_, RK2 derivative, Kan^R^, *mob* ^+^, *tra* ^+^	ATCC
pGPI-SceI	*ori*_R6K_,Tp^R^, *mob* ^+^, carries I-SceI cut site	[37]
pGPI*∆copR*	pGPI-SceI carrying 5’- and 3’-flanking regions of *copR* for mutagenesis of *copR*	This study
pGPI*∆lipA*	pGPI-SceI carrying 5’- and 3’-flanking regions of *lipA* for mutagenesis of *lipA*	This study
pDAI-SceI	*ori*_pBBR1_,Tet^R^, *mob* ^+^, P_*dhfr*_, FLAG epitope, carries I-SceI cut site	[37]
pDA-*copR*	pDAI-SceI with ORF of *copR* replacement of SceI gene	This study
pDA-*lipA*	pDAI-SceI with ORF of *lipA* replacement of SceI gene	This study

### Construction of non-polar deletion mutant strains

Primers used for deletion mutagenesis are listed in Table 2. The unmarked, non-polar mutant strains were constructed as described by Flannagan *et al.* [37], with slight modifications. Briefly, 5’- and 3’-flanking regions of target genes (*copR* and *lipA*) were amplified by PCR from chromosomal DNA, respectively, and the individual PCR products were mixed to generate an in-frame deletion pattern of target genes by an overlapping PCR method, as described previously [38, 39]. Then, the overlapping amplicon containing the in-frame deletion pattern of target genes was sub-cloned into pGPI-SceI, resulting in recombinant plasmids pGPI-*copR* and pGPI-*lipA* respectively. The resulting plasmids were transferred into *B. cenocepacia* by tri-parental mating using *E. coli* HB101 carrying the helper plasmid pRK2013. The single Tp-resistant colonies were then selected as candidates with a single recombination, which was confirmed by PCR. The plasmid pDAI-SceI was introduced by tri-parental conjugation, with the help of pRK2013, to obtain double-crossover event mutants. The pDAI-SceI plasmid was resolved by curing the exconjugants in LB broth. All constructs and mutants were confirmed by PCR analysis and verified by DNA sequencing.

**Table 2:** Primers used in gene deletion experiments

Primers	Sequence (5’ to 3’)^a^
For mutagenesis of *copR*	
LPW23977 (*copR*-UF)	GC**TCTAGA**CCGAAAGGGTTCATTACG
LPW23978 (*copR*-UR)	TCAGCTTGACCTCGAGCCCCTTCTTCAG
LPW23979 (*copR*-DF)	GGGGCTCGAGGTCAAGCTGATCCATACC
LPW23980 (*copR*-DR)	ACAT**GCATGC**TAGCCGTCGAGCAGATC
LPW24342 (*copR*-IF)	GGTTTCAGCGTCGATCTC
LPW24485 (*copR*-IR2)	CATGTCCCATACGTACGA
For mutagenesis of *lipA*	
LPW23981 (*lipA*-UF)	GC**TCTAGA**ACATGCTCGAACGCTGTG
LPW23982 (*lipA*-UR)	CAACGACTGCAATCAGCGCGTTCGATGG
LPW23983 (*lipA*-DF)	CGCGCTGATTGCAGTCGTTGGTCAGTTG
LPW23984 (*lipA*-DR)	C**GAGCTC**AGCACCTGCATGAACAC
LPW24340 (*lipA*-IF)	GGCATACCCGTCTATGTG
LPW24341 (*lipA*-IR)	GACGATGTTTGCCAACTG
For complementation of *∆copR* and *∆lipA*	
LPW29008 (*copR*-CF)	GGAATTC**CATATG**CGGCCATGCGCATCCTGATA
LPW29009 (*copR*-CR)	TGC**TCTAGA**CGTCATGCGTCGTCCTTCGG
LPW29010 (*lipA*-CF)	GGGAATTC**CATATG**CATGAACGTATCGACACGCC
LPW29011 (*lipA*-CR)	CTAG**TCTAGA**TGTGCGGCTACGCCTGATCG

^a^Restriction endonuclease sites in the primer sequences appear in bold

### Complementation of mutant strains

The coding regions of *copR* and *lipA* genes were amplified from chromosomal DNA of AU1054. The PCR products were digested with *Nde*I and *Xba*I and inserted into a similarly digested plasmid, pDAI-SceI [37, 40] resulting in the final constructs of pDA-*copR* and pDA-*lipA*. The complementing plasmids were introduced into the desired mutant strains by tri-parental mating as described above.

### Growth kinetics

To examine the growth kinetics of wild-type (WT) and mutant strains, overnight bacterial cultures of test strains were diluted 1:100 into LB broth and further cultured with agitation (250 rpm) at 37 °C. One ml samples of cell suspension were monitored at different time points by measuring absorbance at 600 nm (OD_600_) using a spectrophotometer as described previously [39]. Experiments were performed in triplicate and repeated three times.

### Cell lines and cell culture

The A549 cell line (American Type Culture Collection) is a human alveolar epithelial carcinoma cell line. A549 cells were grown in F-12 K tissue culture medium supplemented with 10% fetal bovine serum and penicillin/streptomycin 100 U/ml (Gibco) in a humidified atmosphere at 37 °C with 5% CO_2_. For bacterial invasion and replication experiments, A549 cells (2 × 10^5^ cells per well) were seeded in 24-well tissue culture plates (BD Falcon). The cultures were grown at 37 °C with 5% CO_2_. Prior to infection, cells were incubated overnight in antibiotic-free medium.

### Bacterial invasion and intracellular replication assay

The ability of *B. cenocepacia* to invade A549 epithelial cells was examined. Invasion assays were performed by a modification of the gentamicin protection assay described previously [39]. Briefly, A549 cells were infected with mid-log phase (OD_600_ = 0.5) AU1054 at a multiplicity of infection (MOI) of 10. Infected monolayers were centrifuged (300 × *g* for 5 min) and incubated at 37 °C in a humidified atmosphere with 5% CO_2_ for 2 h to allow bacterial entry. Media from the wells were then aspirated and washed three times in phosphate-buffered saline (PBS) to remove unbound bacteria. Extracellular bacteria were then killed by incubation for 2 h in medium containing 2 mg/ml Cef and 2 mg/ml Ami. Cells were washed with PBS, trypsinized and lysed with 0.1% Triton X-100. Intracellular bacteria were quantitated by plating serial dilutions of cell lysates. For intracellular replication assays, after extracellular killing and PBS washing, cells were further incubated in F-12 K medium containing Ami and Cef for 24 h, then trypsinized, lysed and plated to determine the abundance of intracellular bacteria. Bacterial CFUs recovered at 24 h were used to calculate the recovery rate of intracellular replication relative to the baseline values of bacterial invasion at 2 h. Experiments were repeated in triplicate.

### Bacterial adhesion assay

A bacterial adhesion assay was performed as previously described with slight modification [41, 42]. Briefly, A549 cells were seeded into 24-well tissue culture plates at 2 × 10^5^ cells per well and incubated at 37 °C with 5% CO_2_ for 24 h before infection. Bacterial infection of cell lines was as described above for invasion experiments with an MOI of 50:1. Infected cells were incubated for 1 h at 37 °C followed by rinsing five times with PBS to remove non-adherent bacteria. Cells were then trypsinized and lysed with 0.1% Triton X-100 and CFUs were counted by plating serial dilutions of cell lysates on LBA plates.

## Results

### Identified GIs and their characteristics

**Fig. 1:**
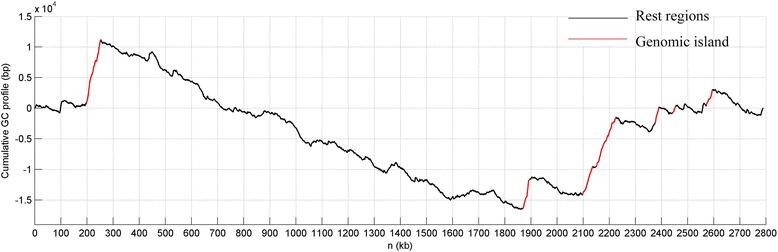
Cumulative GC profile of chromosome II of *B. cenocepacia* AU 1054. All GIs tend to be ascending straight lines (rend colour), indicating they are compositionally homogeneous and AT-richer than the core region

Compared with non-island regions, GIs tend to exhibit more consistent GC contents, reflected by the fact that GI regions usually appear as straight lines within the cumulative GC profile [29, 33]. In addition, an ascension (drop) line within the cumulative GC profile indicates that the region has a lower (higher) GC content than the rest of the host [43]. Through combining the cumulative GC profile method and IslandViewer [29, 35], 14 putative GIs on chromosome I and 7 GIs on chromosome II were identified. However, there are no common predictions between the two methods for chromosome III. Details of the 21 GIs on chromosome I and II are listed in Table 3. All the 21 GIs are AT-rich, given that both chromosomes have average G + C contents of 0.669. As an example, the homogeneity and AT-richness of seven GIs on chromosome II could be illustrated by their patterns within the chromosomal cumulative GC profile (Fig. 1), where all GIs show as ascending straight lines. It was noted there are also several other segments approximating straight lines, but these were filtered out by an additional check of the h index and the IslandViewer tool. Here, all the 21 identified GIs are AT-richer than the core genomes, but they have different bias of G + C extents.

**Table 3:** List of predicted GIs and features. The First column provides position for each GI. The second to ninth column denote the first to the sixth feature of all GIs, Y : Yes and N: No. Both the fifth (VF) and sixth (Repeat sequence) featurres were desribed by two columns

Location	G + C	Integrase /Transposase	tRNA	HHR^1^	Confirmed VF	Putative VF	attL^2^	attR^2^
Chromosome I								
311461.. 333231	0.555	Y	N	N	N	Bcen_0292	1	2
346723.. 356900	0.621	N	Y	Y	N	N	1	1
482937..496366	0.555	N	Y	Y	N	N	4	1
904269..923167	0.626	Y	Y	Y	N	N	0	0
1346581..1357941	0.601	Y	N	N	N	N	0	0
1550123..1571961	0.621	N	N	Y	N	N	0	2
1586748..1648736	0.694	Y	N	N	N	N	0	0
1672260..1684139	0.647	N	N	N	Bcen_1509	N	5	0
1684758..1702085	0.63	Y	N	N	N	N	0	0
1934646..1946682	0.652	Y	Y	N	N	N	0	0
1951043..1963576	0.564	N	Y	Y	N	N	0	1
2805403..2818743	0.576	Y	Y	N	N	N	0	0
2920184..2936821	0.57	Y	Y	Y	N	N	0	0
3158113..3199826	0.591	Y	Y	Y	N	N	1	2
Chromosome II								
198434.. 251490	0.571	Y	N	Y	Bcen_3147	Bcen_3169	16	0
1868507..1893764	0.575	Y	N	Y	N	N	0	0
2097603..2136801	0.61	Y	N	Y		Bcen_4839-Bcen_4842 Bcen_4845-Bcen_4848 Bcen_4850-Bcen_4854	1	0
2148401..2224557	0.616	Y	N	N	N	N	0	0
2375417..2389212	0.586	Y	N	Y	N	N	0	0
2442732..2454392	0.624	Y	N	Y	N	N	0	0
2571116..2595257	0.616	Y	N	Y	N	N	0	0

^1^ HHR: High Ratio of Hypothetical proteins

^2^ attL: Direct Repeat in upstream. attR: Direct Repeat in downstream

As indicated, authentic GIs exhibit certain six specific characteristics. These six characteristics are summarized in Table 3 for all 21 GIs. As can be seen for the feature of G + C bias, GI (1934646..1946682) has the smallest bias of 1.7%, GIs (311461..333231) and (482937..496366) have the largest bias of 11.4%. On average, the bias of the 21 GIs is 6.7%, which approaches significant (Mann–Whitney U test: *U* = 1.67, *p* < 0.10). For the other five characteristics, 16 GIs contain transposases or integrases, eight GIs have flanking tRNA genes, 13 GIs contain high proportions of hypothetical proteins, four GIs contain virulence factor encoding genes, and nine GIs have flanking repeat sequences. In summary, all 21 GIs constitute reliable predictions given that they have multiple features typical of known GIs.

### Putative PAIs and their appearance in other *B. cenocepacia* strains

Virulence factors have been investigated in other strains of *B. cenocepacia* including J2315 and K56-2. From two review [4, 44] and one research articles [45], we obtained hundreds of known virulence factors for *B. cenocepacia*. We identified putative virulence genes based on their consistence of COG ID, i.e. if the gene in AU 1054 belongs to the same COG group with the virulence gene in J2315 and K56-2, the gene will be regarded as putative virulence gene in AU1054. Since COG annotation has been taken as a routine tool of function annotation in prokaryotes, we think that such type transfer of virulence annotation will be much reliable.

By COG match, we obtained a total of 118 putative virulence factors in the strain AU1054 and these are listed in Additional file 1: Table S1. Homologues of these have been experimentally determined to be associated with pathogenesis in another strain of *B. cenocepacia* [4, 44]. Furthermore, 14 genes have been shown to be associated with virulence just in the strain AU1054 by transposon mutagenesis and screening attenuated virulence [45]. Additional file 1: Table S2 lists these confirmed virulence factors. There is only one overlap (Bcen_2776) between the two lists.

With such information from these genes, we could identify which islands are PAIs. Consequently, four GIs are found to contain putative or confirmed virulence factors. Two identified PAIs are located on chromosome I, and they are referred to as PAI 1 (311461..333231) and PAI 2 (1672260..1684139). Regarding the virulence factors, Bcen_0292 is the homologue (identity = 63%) of the experimentally validated VF BCAL3240 in the strain J2315 [4, 44], whereas Bcen_1509 is a validated VF just in the strain AU1054 [45]. Therefore we refer the former as a putative PAI because it contains only the putative virulence factor of AU1054, whereas the latter is a confirmed PAI given that it contains one virulence factor validated as in the strain.

Chromosome II also contains one confirmed PAI (198434..251490) and one putative PAI (2097603..2224557). VF Bcen_3147 has been directly validated [45] and Bcen_3169 is the homologue (identity = 63%) of the validated VF BCAL3299 [4, 44]. Another 13 putative VFs on the second PAI of chromosome II are the homologues (Identity > 60%) of validated VFs BACM326-BACM337 [4, 44]. The two PAIs are referred to as PAI 3 and PAI 4, respectively.

In total, among the 118 putative or confirmed virulence factors, 15 are found to be located in island regions and this means island regions contain 12.7% of all putative virulence factors. However, two of the 14 confirmed virulence factors are located in island regions, corresponding to a ratio of 14.3%. Both ratios are significantly higher (U test: *U* = 2.03, p < 0.05 in the former case and *U* = 6.80, p = 0.000000001 in the latter case) than the percentage (7.4%) of the total size of all islands divided by the total chromosome size. This result is consistent with a previous investigation which showed that GIs regions disproportionately contain more virulence factors than the remainder of a given genome [46].

**Fig. 2:**
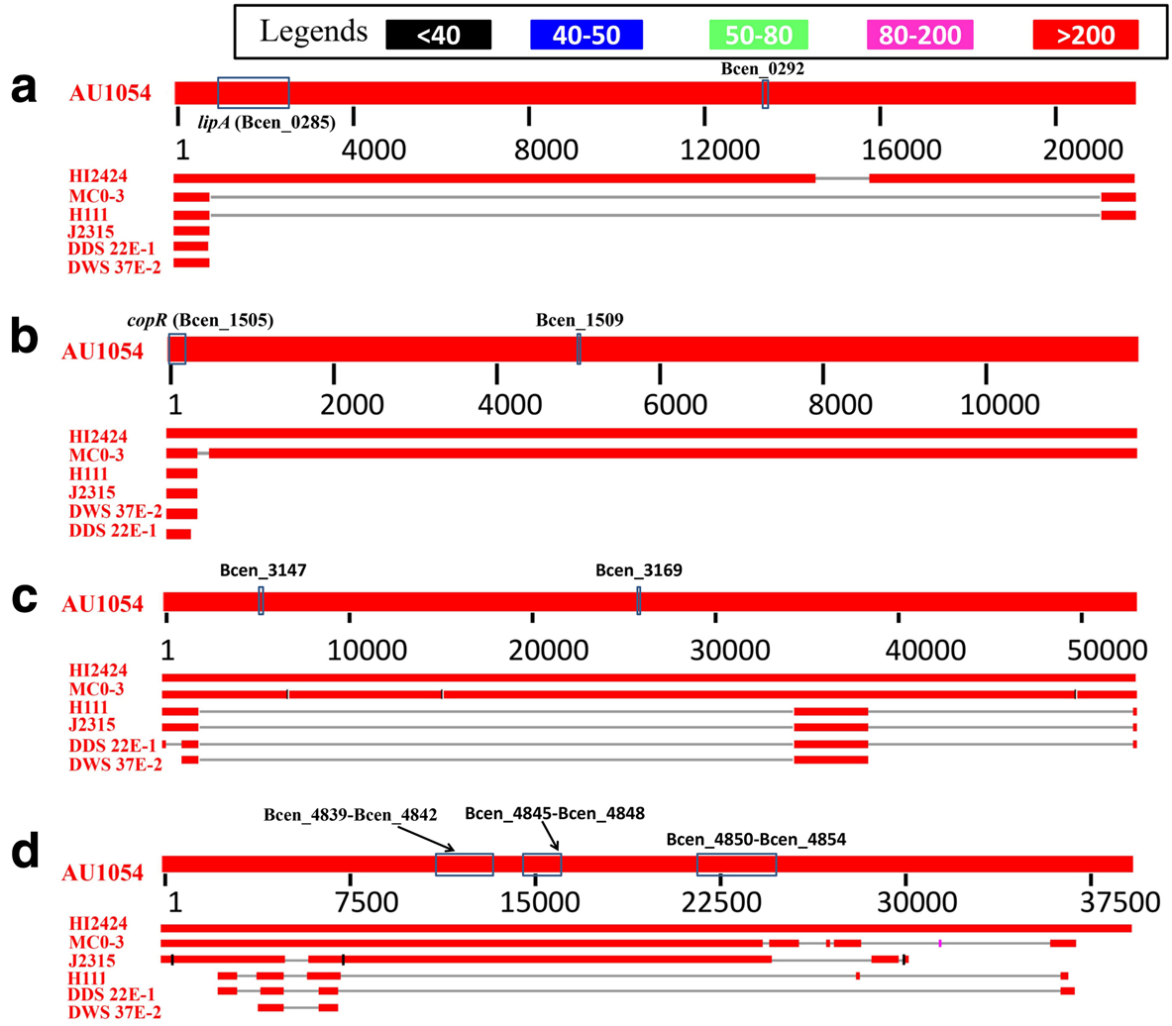
Blast search result of AU 1054 PAIs against genomes in the same species of *B. cenocepacia*. **a**, (**b**), (**c**), and (**d**) Correspond to PAI 1, PAI 2, PAI 3 and PAI 4, respectively. In the four figures only those segments with e-values less than 1e-20 are regarded as effective match. The other six stains are arranged according to match length of their homologous to PAIs in AU1054. That is to say, if a strain has the largest homologous match length, it will be assigned most adjacent with AU 1054. Note that confirmed or putative VFs are marked on the bar of AU 1054 as blue box

The definition of GIs refers to genomic regions present in one strain but absent in closely related strains [24]. Therefore, it is interesting to investigate the appearance of the four PAIs in the other six sequenced strains of *B. cenocepacia*. As Fig. 2 shows, these four PAIs indeed have abnormal phylogenetic distributions if we only consider the homologues with significant match (or hit). For example, PAI 1 shares homologies only in strain HI2424 (Fig. 2a), indicating this strain has the closest evolutionary distance to AU 1054 among the six *B. cenocepacia* strains. PAI 2 exhibits homologues in strains HI2424 and MC0-3 (Fig. 2a), but the former has a higher similarity, indicating MC-03 is the next closest strain to AU1054. PAI 3 reveals a similar case with PAI 2.

### Identification of novel virulence factor determinants or effectors from putative PAIs

**Fig. 3:**
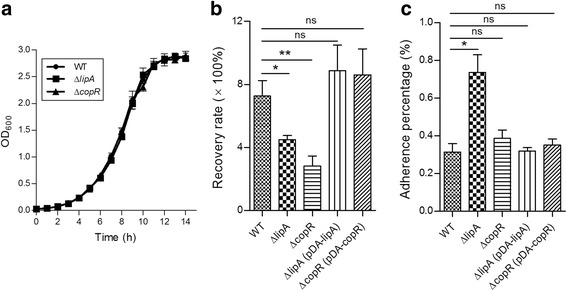
Survival in and adherence to A549 cells of *B. cenocepacia* strains. **a** Growth of WT AU1054 versus mutant strains AU1054∆*lipA* and AU1054∆*copR* cultured in LB. The optical density at OD_600_ was measured hourly over 14 h. **b** Intracellular survival of WT AU1054 and derivative mutant strains in A549 cells. Bacterial infections were performed with MOI of 10, and bacterial survival was represented as recovery rate of CFUs at 24 h relative to that at 2 h. **c** Bacterial adherence assays with different AU1054 strains in A549 cells. Bacterial infections were performed with an MOI of 50. Adherence values were calculated by determining the percentages of bacterial CFUs after adhesion relative to that of original CFUs added for infection (*P < 0.05; **P < 0.01; ***P < 0.001; ns: no significant difference)

In order to further verify our bioinformatics analyses for the PAIs, two putative PAIs were selected for further confirmation with wet experiments. Gene *lipA* (Bcen_0285) from PAI 1, encoding a capsule polysaccharide modification protein, and gene *copR* (Bcen_1505) from PAI 2, encoding a transcriptional regulatory protein, were selected and deleted, respectively. These two genes attracted our attention because that they have not been determined as virulence factors in the species *B. cenocepacia* according to two comprehensive reviews [4, 44], but constitute virulence factors in distant bacteria after our *in silico* comparison with VFDB [47]. We first examined the growth kinetics of the WT AU1054 strain, compared with mutant strains AU1054∆*lipA* and AU1054∆*copR*. As shown in Fig. 3a, the growth rate of each mutant was unaltered in comparison to WT AU1054, indicating that deletion of *lipA* and *copR* genes does not affect bacterial growth rate. We then determined bacterial survival and replication properties of WT AU1054 and mutant strains AU1054∆*lipA* and AU1054∆*copR* in cell line A549, 24 h post-infection. Results showed that mutants with *lipA* and *copR* deletion had a significantly lower intracellular multiplication than that of WT AU1054. When the mutant strains were complemented with pDA-*lipA* and pDA-*copR* respectively, their intracellular replication abilities were fully restored (Fig. 3b), confirming that *lipA* and *copR* genes play a significant role for bacterial survival and replication in human cell lines. The importance of the genes *lipA* (from PAI 1) and *copR* (from PAI 2) for bacterial invasion and replication indicates that they are required for full invasiveness of *B. cenocepacia* and may function as virulence determinants for bacterial pathogenesis and host infection.

Furthermore, there is evidence that the bacterial capsule could affect adhesion to host cells, influence the elimination by human neutrophils, and modulate the virulence in animal models of infection [48, 49]. In order to determine if the observed replication and invasion defect of the mutants was due to reduced binding to A549 cells, we performed adhesion assays employing WT and mutant strains and compared their adherence characteristics. Consistent with the contribution observed for the capsule in other bacteria for intracellular invasion [48], capsule mutant strain AU1054∆*lipA* was approximately two-fold higher in adherence capacity to human cells than the encapsulated strains, including the WT and mutant strain AU1054∆*copR*. The adherence of AU1054∆*lipA* could be compromised in comparison to that of WT strain after complementation with pDA-*lipA* (Fig. 3c), indicating that the capsule negatively affects AU1054 adhesion to human epithelial cells. Taken together, although capsule-deficient mutants were internalized much more efficiently than that of encapsulated bacteria, their invasion and replication abilities were much lower in infected cells, suggesting that the capsule contributes to the establishment of bacterial infection.

Two novel virulence-related genes *lipA* from PAI 1 and *copR* from PAI 2 were confirmed by wet experiments. Sequences of the two genes were extracted from ‘.ffn’ file. BLASTn search was performed via NCBI blast web server (https://blast.ncbi.nlm.nih.gov/Blast.cgi) with optional organism ‘*Burkholderia cenocepacia* (taxid:95486)’ and found that gene *lipA* has homologous gene in strain HI2424. Using the same method, we identified another six homologous genes of *copR* in strain J2315, H111, HI2424, MC0-3, DDS 22E-1, DWS 37E-2. In order to verify whether the homologous genes with *lipA* and *copR* are located in island region or the core genome in the other six strains, we also identified islands for them. Consequently, the homologous gene of *lipA*, Bcen2424_0769, is located with genomic island (864830..881195) in strain HI2424. Conversely, all the homologous genes of *copR* are located in the core genomes of the six strains. Specifically, the PAI 1 located in the chromosome I of strain AU1504 contains genes Bcen_0284-Bcen_0285 and Bcen_0287-Bcen_0298. Among them, Bcen_0285 corresponds to *lipA* and its function is capsule polysaccharide biosynthesis PAI 1. Furthermore, through manually inspecting the annotation file, we found that PAI 1 carry five additional genes (Bcen_287, Bcen_292, Bcen_293, Bcen_295, Bcen_296) for capsule polysaccharide biosynthesis.

## Discussion

GIs are genomic regions that reveal evidence of horizontal DNA transfer, particularly in bacteria [10]. GIs can code for many functions, symbiotic or pathogenic, and may augment an organism’s adaptation to the host or environment [50]. Two steps were involved with GI integration and formation [51]. Previously, GIs were found to disproportionately contain more virulence factors than the rest of a given genome [46]. Here, the ratios of virulence factors in island regions and in the remaining genome in the strain *B. cenocepacia* AU 1054 are 12.7% and 7.4%, respectively, consistent with previous observations. Furthermore, GIs tend to contain distinguishing characteristics, such as limited length range, distinct composition, mobility genes, flanking tRNA and higher ratios of hypothetical genes [25, 26]. After thorough analysis, all 21 GIs identified here share at least one conserved feature, suggesting that the predictions are highly reliable. However, a high rate of false-positive predictions is a widely reported phenomenon using the existing GI predicting methods. To compare the result of combining the two methods and only retaining the common predictions, we also analyzed features of GIs exclusively predicted by the cumulative GC profile or IsandViewer tool. As shown in Additional file 1: Tables S3 and S4, four of the 16 GIs exclusively predicted by the former method are GC richer than the host, and four also have higher GC contents among the 22 GIs exclusively predicted by the latter method. The property of GC richness decreases the possibility that they constitute genuine predictions. As for the other five typical features, 21 GIs share 2.24 common features on average, whereas 38 exclusive GIs only exhibit 1.29 on average. The difference between the two average numbers is statistically significant (*p* = 0.0007 by student’s t-test). Therefore, the approach of coupling two independent computational methods and selecting common predictions has been shown to be successful, and the final predictions do indeed have lower false-positives.

Four GIs were identified as putative PAIs by combining island-prediction tools and identification of putative virulence factors in strain AU 1054. Although these genes have been validated as enhancing pathogenicity in other strains of *B. cenocepacia*, they are not validated in AU 1054, hence the islands are termed ‘putative’ PAIs. With bioinformatics tools, we identified two further potential virulence factors in two of the putative PAIs. These factors were not previously validated in *B. cenocepacia* but have been validated in distantly related species. Using knock-out experiments in viable host cells, we demonstrated the role of these factors to favor infection. Our strategy of jointing two tools may be used to identifying GIs in other bacterial genomes. We downloaded the 13 GIs of the strain *B. cenocepacia* J2315 identified by comparative genomics method [23], and use this dataset as the gold standard, 9 of them were also identified by our combining methods of cumulative GC plot and the Island Viewer. We obtained precision value of 42.86% for the Cumulative GC profile [52], whereas IslandViewer [29, 35] has the precision of 12.63%. After combing the two methods, the precision increases to 50%. Following this approach, we propose a convenient and rapid pipeline to identify novel virulence factors in certain pathogenic strains. First, GIs can be rapidly identified using computational techniques. Second, DNA sequence homology searches of the genes contained in GI regions can identify possible virulence factors. Finally, gene deletion experiments may validate (or otherwise) the function of the predicted virulence factors. Because these genes contribute to pathogenicity in distantly-related species, and that virulence factors are frequently associated with GIs [46], the predicted virulence factors in island regions have much-elevated likelihood to authentically contribute to infection.

## Conclusions

In this work, we identified 21 (GIs) in *B. cenocepacia* strain AU 1054 by combining two computational tools. Feature analyses suggest that the predictions are reliable and hence illustrate the advantage of joint predictions by two independent methods. Four GIs were further identified as PAIs because they contain putative virulence factors. Two PAIs were confirmed by experimental validation of virulence related functions for genes in them. Such approach of theoretically predicting GIs, and then identifying potential virulence factors in the island regions with final validation using wet experiments may be used to discover or validate virulence factors in other bacterial species and strains.

## Additional file

Additional file 1:
**Table S1.** List of putative virulence factors in AU 1054 generated by COG ID match with known VFs in the strain J2315, and information of known virulence factors in the J2315 were obtained from Ref. 4 and 48. **Table S2.** List of virulence factors identified in AU 1054 by screening attenuated virulence, which is extracted from Ref. 49. **Table S3.** Feature analysis of 16 GIs exclusively predicted by the cumulative GC profile. **Table S4.** Feature analysis of 22 GIs exclusively predicted by the IslandViewer tool. (DOCX 46 kb)

## References

[1] Medina-Pascual MJ, Valdezate S, Carrasco G, Villalon P, Garrido N, Saez-Nieto JA (2015). Increase in isolation of *Burkholderia* contaminans from Spanish patients with cystic fibrosis. Clin Microbiol Infect.

[2] Regan KH, Bhatt J (2014). Eradication therapy for *Burkholderia cepacia* complex in people with cystic fibrosis. Cochrane Database Syst Rev.

[3] Parkins MD, Floto RA (2015). Emerging bacterial pathogens and changing concepts of bacterial pathogenesis in cystic fibrosis. J Cyst Fibros.

[4] Drevinek P, Mahenthiralingam E (2010). *Burkholderia cenocepacia* in cystic fibrosis: epidemiology and molecular mechanisms of virulence. Clin Microbiol Infect.

[5] McDowell A, Mahenthiralingam E, Dunbar KE, Moore JE, Crowe M, Elborn JS (2004). Epidemiology of *Burkholderia cepacia* complex species recovered from cystic fibrosis patients: issues related to patient segregation. J Med Microbiol.

[6] de Vrankrijker AM, Wolfs TF, van der Ent CK (2010). Challenging and emerging pathogens in cystic fibrosis. Paediatr Respir Rev.

[7] Mahenthiralingam E, Bischof J, Byrne SK, Radomski C, Davies JE, Av-Gay Y, Vandamme P (2000). DNA-Based diagnostic approaches for identification of *Burkholderia cepacia* complex, *Burkholderia vietnamiensis*, *Burkholderia multivorans*, *Burkholderia stabilis*, and *Burkholderia cepacia* genomovars I and III. J Clin Microbiol.

[8] Eusebio N, Coutinho CP, Sa-Correia I, Araujo R (2013). SNaPBcen: a novel and practical tool for genotyping *Burkholderia cenocepacia*. J Clin Microbiol.

[9] Pretto L, De-Paris F, Mombach Pinheiro Machado AB, Francisco Martins A, Barth AL (2013). Genetic similarity of *Burkholderia cenocepacia* from cystic fibrosis patients. Braz J Infect Dis.

[10] Mahenthiralingam E, Urban TA, Goldberg JB (2005). The multifarious, multireplicon *Burkholderia cepacia* complex. Nat Rev Microbiol.

[11] Coenye T, LiPuma JJ (2002). Multilocus restriction typing: a novel tool for studying global epidemiology of *Burkholderia cepacia* complex infection in cystic fibrosis. J Infect Dis.

[12] Tatusova T, Ciufo S, Fedorov B, O’Neill K, Tolstoy I (2015). RefSeq microbial genomes database: new representation and annotation strategy. Nucleic Acids Res.

[13] Bartell JA, Yen P, Varga JJ, Goldberg JB, Papin JA (2014). Comparative metabolic systems analysis of pathogenic *Burkholderia*. J Bacteriol.

[14] Juhas M, Stark M, von Mering C, Lumjiaktase P, Crook DW, Valvano MA, Eberl L (2012). High confidence prediction of essential genes in *Burkholderia cenocepacia*. PLoS One.

[15] Lynch KH, Stothard P, Dennis JJ (2012). Comparative analysis of two phenotypically-similar but genomically-distinct *Burkholderia cenocepacia*-specific bacteriophages. BMC Genomics.

[16] Passot FM, Calderon V, Fichant G, Lane D, Pasta F (2012). Centromere binding and evolution of chromosomal partition systems in the *Burkholderiales*. J Bacteriol.

[17] Mira NP, Madeira A, Moreira AS, Coutinho CP, Sa-Correia I (2011). Genomic expression analysis reveals strategies of *Burkholderia cenocepacia* to adapt to cystic fibrosis patients’ airways and antimicrobial therapy. PLoS One.

[18] Graindorge A, Menard A, Monnez C, Cournoyer B (2012). Insertion sequence evolutionary patterns highlight convergent genetic inactivations and recent genomic island acquisitions among epidemic *Burkholderia cenocepacia*. J Med Microbiol.

[19] Sass A, Marchbank A, Tullis E, LiPuma JJ, Mahenthiralingam E (2011). Spontaneous and evolutionary changes in the antibiotic resistance of *Burkholderia cenocepacia* observed by global gene expression analysis. BMC Genomics.

[20] Guo FB, Ning LW, Huang J, Lin H, Zhang HX (2010). Chromosome translocation and its consequence in the genome of *Burkholderia cenocepacia* AU-1054. Biochem Biophys Res Commun.

[21] Morrow JD, Cooper VS (2012). Evolutionary effects of translocations in bacterial genomes. Genome Biol Evol.

[22] diCenzo G, Milunovic B, Cheng J, Finan TM (2013). The tRNA^arg^ gene and *engA* are essential genes on the 1.7-Mb pSymB megaplasmid of *Sinorhizobium meliloti* and were translocated together from the chromosome in an ancestral strain. J Bacteriol.

[23] Holden MT, Seth-Smith HM, Crossman LC, Sebaihia M, Bentley SD, Cerdeno-Tarraga AM, Thomson NR, Bason N, Quail MA, Sharp S (2009). The genome of *Burkholderia cenocepacia* J2315, an epidemic pathogen of cystic fibrosis patients. J Bacteriol.

[24] Dobrindt U, Hochhut B, Hentschel U, Hacker J (2004). Genomic islands in pathogenic and environmental microorganisms. Nat Rev Microbiol.

[25] Vernikos GS, Parkhill J (2008). Resolving the structural features of genomic islands: a machine learning approach. Genome Res.

[26] Hsiao WW, Ung K, Aeschliman D, Bryan J, Finlay BB, Brinkman FS (2005). Evidence of a large novel gene pool associated with prokaryotic genomic islands. PLoS Genet.

[27] Ou HY, He XY, Harrison EM, Kulasekara BR, Bin Thani A, Kadioglu A, Lory S, Hinton JCD, Barer MR, Deng ZX (2007). MobilomeFINDER: web-based tools for *in silico* and experimental discovery of bacterial genomic islands. Nucleic Acids Res.

[28] Guo FB, Xia ZK, Wei W, Zhao HL (2014). Statistical analyses of conserved features of genomic islands in bacteria. Genet Mol Res.

[29] Zhang R, Zhang CT (2004). A systematic method to identify genomic islands and its applications in analyzing the genomes of *Corynebacterium glutamicum* and *Vibrio vulnificus* CMCP6 chromosome I. Bioinformatics.

[30] Zhang CT, Zhang R (2004). Genomic islands in *Rhodopseudomonas palustris*. Nat Biotechnol.

[31] Wei W, Guo F (2011). Prediction of genomic islands in seven human pathogens using the Z-island method. Genet Mol Res.

[32] Guo FB, Wei W (2012). Prediction of genomic islands in three bacterial pathogens of pneumonia. Int J Mol Sci.

[33] Zhang R, Ou HY, Gao F, Luo H (2014). Identification of Horizontally-transferred Genomic Islands and Genome Segmentation Points by Using the GC Profile Method. Curr Genomics.

[34] Langille MG, Brinkman FS (2009). IslandViewer: an integrated interface for computational identification and visualization of genomic islands. Bioinformatics.

[35] Dhillon BK, Laird MR, Shay JA, Winsor GL, Lo R, Nizam F, Pereira SK, Waglechner N, McArthur AG, Langille MG (2015). IslandViewer 3: more flexible, interactive genomic island discovery, visualization and analysis. Nucleic Acids Res.

[36] Langille MG, Hsiao WW, Brinkman FS (2010). Detecting genomic islands using bioinformatics approaches. Nat Rev Microbiol.

[37] Flannagan RS, Linn T, Valvano MA (2008). A system for the construction of targeted unmarked gene deletions in the genus *Burkholderia*. Environ Microbiol.

[38] Xiong L, Teng JL, Watt RM, Kan B, Lau SK, Woo PC (2014). Arginine deiminase pathway is far more important than urease for acid resistance and intracellular survival in *Laribacter hongkongensis*: a possible result of *arc* gene cassette duplication. BMC Microbiol.

[39] Xiong L, Teng JL, Watt RM, Liu C, Lau SK, Woo PC (2015). Molecular characterization of arginine deiminase pathway in *Laribacter hongkongensis* and unique regulation of arginine catabolism and anabolism by multiple environmental stresses. Environ Microbiol.

[40] Aubert DF, Flannagan RS, Valvano MA (2008). A novel sensor kinase-response regulator hybrid controls biofilm formation and type VI secretion system activity in *Burkholderia cenocepacia*. Infect Immun.

[41] Gupta R, Yang J, Dong Y, Swiatlo E, Zhang JR, Metzger DW, Bai G (2013). Deletion of *arcD* in *Streptococcus pneumoniae* D39 impairs its capsule and attenuates virulence. Infect Immun.

[42] Tomich M, Herfst CA, Golden JW, Mohr CD (2002). Role of flagella in host cell invasion by *Burkholderia cepacia*. Infect Immun.

[43] Zhang CT, Wang J, Zhang R (2001). A novel method to calculate the G + C content of genomic DNA sequences. J Biomol Struct Dyn.

[44] Loutet SA, Valvano MA (2010). A decade of *Burkholderia cenocepacia* virulence determinant research. Infect Immun.

[45] Somvanshi VS, Viswanathan P, Jacobs JL, Mulks MH, Sundin GW, Ciche TA (2010). The type 2 secretion pseudopilin, *gspJ*, is required for multihost pathogenicity of *Burkholderia cenocepacia* AU1054. Infect Immun.

[46] Ho Sui SJ, Fedynak A, Hsiao WW, Langille MG, Brinkman FS (2009). The association of virulence factors with genomic islands. PLoS One.

[47] Chen L, Xiong Z, Sun L, Yang J, Jin Q (2012). VFDB 2012 update: toward the genetic diversity and molecular evolution of bacterial virulence factors. Nucleic Acids Res.

[48] Spinosa MR, Progida C, Tala A, Cogli L, Alifano P, Bucci C (2007). The *Neisseria meningitidis* capsule is important for intracellular survival in human cells. Infect Immun.

[49] Geisinger E, Isberg RR (2015). Antibiotic modulation of capsular exopolysaccharide and virulence in *Acinetobacter baumannii*. PLoS Pathog.

[50] Dutta C, Paul S (2012). Microbial lifestyle and genome signatures. Curr Genomics.

[51] Bellanger X, Payot S, Leblond-Bourget N, Guedon G (2014). Conjugative and mobilizable genomic islands in bacteria: evolution and diversity. FEMS Microbiol Rev.

[52] Wei W, Gao F, Du MZ, Hua HL, Wang J, Guo FB. Zisland Explorer: detect genomic islands by combining homogeneity and heterogeneity properties. Brief Bioinform. 2017. doi:10.1093/bib/bbw019. 10.1093/bib/bbw019 PMC5429010 26992782

[53] Xiong L, Yang Y, Ye YN, Teng JL, Chan E, Watt RM, Guo FB, Lau SK, Woo PC. *Laribacter hongkongensis* anaerobic adaptation mediated by arginine metabolism is controlled by the cooperation of FNR and ArgR. Environ Microbiol. 2017;19(3):1266-80. 10.1111/1462-2920.13657 28028888

