# Supplementary material for: Identification and analysis of genomic islands in Burkholderia cenocepacia AU 1054 with emphasis on pathogenicity islands
Source: BMC Microbiol. 2017 Mar 27;17:73. doi: 10.1186/s12866-017-0986-6 (PMC5369199; doi:10.1186/s12866-017-0986-6)
Supplement: Additional file 1: — Table S1. List of putative virulence factors in AU 1054 generated by COG ID match with known VFs in the strain J2315, and information of known virulence factors in the J2315 were obtained from Ref. 4 and 48. Table S2. List of virulence factors identified in AU 1054 by screening attenuated virulence, which is extracted from Ref. 49. Table S3. Feature analysis of 16 GIs exclusively predicted by the cumulative GC profile. Table S4. Feature analysis of 22 GIs exclusively predicted by the IslandViewer tool. (DOCX 46 kb) [file 12866_2017_986_MOESM1_ESM.docx]

**Additional file 1**

**Table S1** List of putative virulence factors in AU 1054 generated by homology transfer from the strain J2315, and information of known virulence factors in the J2315 were obtained from Ref. 4 and 44.

| Synonym Code | Gene | Product/Function |  |  |
| --- | --- | --- | --- | --- |
| Bcen_0242 | - | catalase/peroxidase HPI |  |  |
| Bcen_0292 | - | ABC transporter |  | |
| Bcen_0392 | - | dTDP-4-dehydrorhamnose reductase |  |  |
| Bcen_0405 | - | glycosyl transferase family protein |  |  |
| Bcen_0406 | - | NAD-dependent epimerase/dehydratase |  |  |
| Bcen_0408 | - | polysaccharide biosynthesis protein CapD |  |  |
| Bcen_0415 | - | lipopolysaccharide heptosyltransferase I |  |  |
| Bcen_0573 | - | bifunctional ADP-heptose synthase |  |  |
| Bcen_0574 | - | ADP-L-glycero-D-manno-heptose-6-epimerase |  |  |
| Bcen_0644 | *acpP* | acyl carrier protein |  | |
| Bcen_0647 | - | RNA polymerase sigma factor RpoE |  |  |
| Bcen_0650 | - | peptidase S1C, Do |  | |
| Bcen_1049 | - | sigma-54 dependent trancsriptional regulator |  |  |
| Bcen_1142 | - | periplasmic multidrug efflux lipoprotein precursor |  |  |
| Bcen_1143 | - | multidrug efflux protein |  |  |
| Bcen_1144 | - | RND efflux system, outer membrane lipoprotein, NodT |  |  |
| Bcen_1152 | - | extracytoplasmic-function sigma-70 factor |  |  |
| Bcen_1153 | - | MbtH-like protein |  | |
| Bcen_1154 | - | taurine catabolism dioxygenase TauD/TfdA |  |  |
| Bcen_1155 | - | ABC transporter |  | |
| Bcen_1156 | - | iron-hydroxamate transporter permease subunit |  |  |
| Bcen_1157 | - | ferric iron reductase |  | |
| Bcen_1158 | - | periplasmic binding protein |  |  |
| Bcen_1159 | - | cyclic peptide transporter |  |  |
| Bcen_1160 | - | amino acid adenylation |  |  |
| Bcen_1161 | - | amino acid adenylation |  |  |
| Bcen_1162 | - | hypothetical protein |  | |
| Bcen_1163 | - | lysine/ornithine N-monooxygenase |  |  |
| Bcen_1164 | - | TonB-dependent siderophore receptor |  |  |
| Bcen_1165 | - | folate-dependent phosphoribosylglycinamide formyltransferase PurN-like protein |  |  |
| Bcen_1166 | - | hypothetical protein |  | |
| Bcen_1233 | - | propeptide, peptidase M4 and M36 |  |  |
| Bcen_1823 | - | superoxide dismutase, copper/zinc binding |  |  |
| Bcen_1997 | - | RND efflux system, outer membrane lipoprotein, NodT |  |  |
| Bcen_1998 | - | hydrophobe/amphiphile efflux-1 HAE1 |  |  |
| Bcen_1999 | - | secretion protein HlyD |  |  |
| Bcen_2005 | - | hypothetical protein |  | |
| Bcen_2006 | - | peptidase S1C, Do |  | |
| Bcen_2177 | - | RNA polymerase factor sigma-54 |  |  |
| Bcen_2354 | - | porin |  |  |
| Bcen_2451 | *fliG* | flagellar motor switch protein G |  |  |
| Bcen_2453 | - | flagellar protein export ATPase FliI |  |  |
| Bcen_2454 | - | flagellar export FliJ |  | |
| Bcen_2620 | - | ImcF-related |  | |
| Bcen_2621 | - | hypothetical protein |  | |
| Bcen_2622 | - | OmpA/MotB protein |  | |
| Bcen_2631 | - | ImpA domain-containing protein |  |  |
| Bcen_2632 | - | ATPase AAA |  | |
| Bcen_2634 | - | hypothetical protein |  | |
| Bcen_2635 | - | hypothetical protein |  | |
| Bcen_2636 | - | hypothetical protein |  | |
| Bcen_2637 | - | hypothetical protein |  | |
| Bcen_2638 | - | hypothetical protein |  | |
| Bcen_2639 | - | hypothetical protein |  | |
| Bcen_2640 | - | hypothetical protein |  | |
| Bcen_2641 | - | hypothetical protein |  | |
| Bcen_2642 | - | hypothetical protein |  | |
| Bcen_2643 | - | hypothetical protein |  | |
| Bcen_2776 | *paaA* | phenylacetate-CoA oxygenase subunit PaaA |  |  |
| Bcen_2780 | - | phenylacetate-CoA oxygenase/reductase subunit PaaK |  |  |
| Bcen_2785 | - | 4-hydroxyphenylpyruvate dioxygenase |  |  |
| Bcen_2866 | - | flagellin |  |  |
| Bcen_3169 | - | catalase/peroxidase HPI |  |  |
| Bcen_3302 | - | propeptide, peptidase M4 and M36 |  |  |
| Bcen_3374 | - | TonB-dependent siderophore receptor |  |  |
| Bcen_3443 | - | two component transcriptional regulator |  |  |
| Bcen_3510 | - | type III secretion FHIPEP |  |  |
| Bcen_3511 | - | Type III secretion protein HrpO |  |  |
| Bcen_3512 | - | Type III secretion system outer membrane pore YscC/HrcC |  |  |
| Bcen_3513 | - | Type III secretion system YscD/HrpQ |  |  |
| Bcen_3514 | - | hypothetical protein |  | |
| Bcen_3515 | - | hypothetical protein |  | |
| Bcen_3516 | - | Type III secretion system lipoprotein HrcJ/YscJ |  |  |
| Bcen_3517 | - | hypothetical protein |  | |
| Bcen_3518 | - | Type III secretion system HrpE/YscL |  |  |
| Bcen_3519 | - | ATP synthase FliI/YscN |  |  |
| Bcen_3520 | - | mucin-associated surface protein |  |  |
| Bcen_3521 | - | Type III secretion protein SpaR/YscT |  |  |
| Bcen_3522 | - | type III secretion exporter |  |  |
| Bcen_3641 | - | autoinducer synthesis protein |  |  |
| Bcen_3643 | - | LuxR family transcriptional regulator |  |  |
| Bcen_3644 | - | MgtC/SapB transporter |  |  |
| Bcen_3678 | - | amidase |  |  |
| Bcen_4443 | - | peptidase S8/S53 subtilisin kexin sedolisin |  |  |
| Bcen_4539 | - | protein-tyrosine kinase |  |  |
| Bcen_4541 | - | protein tyrosine phosphatase |  |  |
| Bcen_4544 | - | mannose-1-phosphate guanylyltransferase/mannose-6-phosphate isomerase |  |  |
| Bcen_4789 | - | acyltransferase 3 |  | |
| Bcen_4800 | - | periplasmic sensor hybrid histidine kinase |  |  |
| Bcen_4839 | - | plasmid-like protein |  | |
| Bcen_4840 | - | hypothetical protein |  | |
| Bcen_4841 | - | hypothetical protein |  | |
| Bcen_4842 | - | hypothetical protein |  | |
| Bcen_4845 | - | type II secretion system protein E |  |  |
| Bcen_4846 | - | conjugation TrbI-like protein |  |  |
| Bcen_4847 | - | conjugal transfer protein TrbG/VirB9/CagX |  |  |
| Bcen_4848 | - | virulence protein |  | |
| Bcen_4850 | - | TrbL/VirB6 plasmid conjugal transfer protein |  |  |
| Bcen_4851 | - | type IV secretion/conjugal transfer ATPase |  |  |
| Bcen_4852 | - | type IV secretory pathway, VirB3-like |  |  |
| Bcen_4853 | - | hypothetical protein |  | |
| Bcen_4854 | - | lytic transglycosylase, catalytic |  |  |
| Bcen_4980 | - | LuxR family transcriptional regulator |  |  |
| Bcen_5187 | - | two component LuxR family transcriptional regulator |  |  |
| Bcen_5425 | - | hypothetical protein |  | |
| Bcen_5427 | *hmuV* | hemin importer ATP-binding subunit |  |  |
| Bcen_5510 | - | hypothetical protein |  | |
| Bcen_5599 | - | secretion protein HlyD |  |  |
| Bcen_5600 | - | hydrophobe/amphiphile efflux-1 HAE1 |  |  |
| Bcen_5601 | - | RND efflux system, outer membrane lipoprotein, NodT |  |  |
| Bcen_5634 | - | LysR family transcriptional regulator |  |  |
| Bcen_6216 | - | polysaccharide deacetylase |  |  |
| Bcen_6217 | - | NAD-dependent epimerase/dehydratase family protein |  |  |
| Bcen_6218 | - | formyltransferase |  | |
| Bcen_6219 | - | glycosyl transferase family protein |  |  |
| Bcen_6220 | - | DegT/DnrJ/EryC1/StrS aminotransferase |  |  |
| Bcen_6221 | - | hypothetical protein |  | |
| Bcen_6222 | - | glycosyl transferase family protein |  |  |

| Synonym Code | Gene | Product/Function |
| --- | --- | --- |
| Bcen_0002 | *-* | sodium/hydrogen exchanger |
| Bcen_0015 | *-* | general secretion pathway protein J |
| Bcen_0171 | *leuS* | leucyl-tRNA synthetase |
| Bcen_0566 | *-* | 3-phosphoshikimate 1-carboxyvinyltransferase |
| Bcen_1489 | *-* | LysR family transcriptional regulator |
| Bcen_1509 | *-* | hypothetical protein |
| Bcen_2123 | *-* | FAD linked oxidase-like protein |
| Bcen_2245 | *-* | UBA/THIF-type NAD/FAD binding fold |
| Bcen_2776 | *paaA* | phenylacetate-CoA oxygenase subunit PaaA |
| Bcen_3021 | *-* | hypothetical protein |
| Bcen_3147 | *-* | AAA ATPase |
| Bcen_4402 | *-* | amidophosphoribosyltransferase |
| Bcen_4409 | *-* | tryptophan synthase subunit beta |
| Bcen_4651 | *-* | argininosuccinate synthase |

**Table S2** List of virulence factors identified in AU 1054 by screening attenuated virulence, which are extracted from Ref. 45.

**Table S3**. Feature analysis of 16 GIs exclusively predicted by the cumulative GC profile

| Location | Size | G+C | Feature | Confirmed VF | Putative VF |
| --- | --- | --- | --- | --- | --- |
| Chromosome I |  |  |  |  |  |
| 47514..69771 | 22258 | 0.608 | Hypothetical, RNA | - | - |
| 425350.. 450500 | 25151 | 0.627 | Transposase, VF | - | Bcen_0392, Bcen_0405, Bcen_0406, Bcen_0408 |
| 1184700.. 1198600 | 13901 | 0.594 | Integrase, RNA, hypothetical | - | - |
| 1255100.. 1280700 | 25601 | 0.717 | - | - | - |
| 1453100.. 1544100 | 91001 | 0.702 | Hypothetical | - | - |
| 1781300.. 1843000 | 61701 | 0.639 | RNA | - | - |
| 2995900.. 3054400 | 58501 | 0.615 | RNA, VF | Bcen_2776 |  |
| Chromosome II |  |  |  |  |  |
| 94355.. 101610 | 7256 | 0.567 | RNA | - | - |
| 428230.. 437900 | 9671 | 0.640 | - | - | - |
| 975000.. 1040300 | 65301 | 0.695 | Transposase, integrase, IS | - | - |
| Chromosome Ⅲ |  |  |  |  |  |
| 83226.. 90968 | 7743 | 0.601 | RNA, hypothetical | - | - |
| 369680.. 383230 | 13551 | 0.610 | Hypothetical | - | - |
| 416130.. 448060 | 31931 | 0.623 | Transposase, IS, RNA, hypothetical | - | - |
| 750970.. 759680 | 8711 | 0.625 | RNA | - | - |
| 995810.. 1005500 | 9691 | 0.615 | - | - | - |
| 1182600.. 1194200 | 11601 | 0.725 | - | - | - |

**Table S4** Feature analysis of 22 GIs exclusively predicted by the IslandViewer tool

| Location | Size | G+C | Feature | Confirmed VF | Putative VF |
| --- | --- | --- | --- | --- | --- |
| Chromosome I |  |  |  |  |  |
| 441794..447666 | 5872 | 0.6106 | Hypothetical | - | - |
| 1185989..1196110 | 10121 | 0.5789 | Integrase, RNA, hypothetical | - | - |
| 1533107..1541019 | 7912 | 0.6814 | Hypothetical | - | - |
| 1659704..1664109 | 4405 | 0.6607 | - | - | - |
| 1709122..1713309 | 4187 | 0.6483 | - | - | - |
| 1713529..1719103 | 5574 | 0.6778 | Hypothetical | - | - |
| 1731830..1737067 | 5237 | 0.7058 | - | - | - |
| 1748933..1755645 | 6712 | 0.6598 | - | - | - |
| Chromosome II |  |  |  |  |  |
| 200868..225403 | 24535 | 0.5577 | Integrase, hypothetical, VF | Bcen_3147 | - |
| 237573..250723 | 13150 | 0.5476 | Hypothetical | - | - |
| 433352..440077 | 6725 | 0.6029 | Hypothetical | - | - |
| 527218..531714 | 4496 | 0.5726 | Hypothetical | - | - |
| 2553488..2561921 | 8433 | 0.5767 | Hypothetical, IS, transposase | - | - |
| 2780614..2786123 | 5509 | 0.594 | - | - | - |
| Chromosome Ⅲ |  |  |  |  |  |
| 62076..72756 | 10680 | 0.6481 | Transposase, IS | - | - |
| 84817..91442 | 6625 | 0.5836 | RNA, hypothetical | - | - |
| 109210..118789 | 9579 | 0.6463 | Hypothetical, transposase, IS, VF | - | Bcen_5599 |
| 373036..384036 | 11000 | 0.6019 | Hypothetical | - | - |
| 595413..600345 | 4932 | 0.674 | Hypothetical, RNA | - | - |
| 635654..648886 | 13232 | 0.6785 | Hypothetical, transposase, IS | - | - |
| 749121..763214 | 14093 | 0.6496 | RNA | - | - |
| 835838..843154 | 7316 | 0.6676 | - | - | - |
